# *Rickettsia heilongjiangensis* suppresses RIPK1 kinase-mediated host cell death during the infection

**DOI:** 10.1128/iai.00158-25

**Published:** 2025-07-03

**Authors:** Maozhang He, Qingyin Shi, Yu Xin, Shurui Zheng, Yan Liu, Kehan Xu

**Affiliations:** 1 Anhui Province Key Laboratory of Zoonoses, Department of Microbiology, School of Basic Medical Sciences, Anhui Medical University 659392, Hefei, People’s Republic of China; Washington State University, Pullman, Washington, USA

**Keywords:** *Rickettsia*, PANoptosis, receptor interacting protein kinase 1 (RIPK1), tumor necrosis factor (TNF), caspase

## Abstract

Spotted fever group *Rickettsia* (SFGR) poses a significant challenge in the field of tick-borne diseases, characterized by its obligate intracellular lifestyle and its ability to disrupt various host cellular pathways. A deeper understanding of *Rickettsia*’s interactions with immune signaling is crucial for the development of novel therapeutic strategies. While previous research has shown that SFGR infection manipulates host cell death responses, the specific effects on receptor-interacting protein kinase 1 (RIPK1)-mediated multiple cell death pathways—collectively referred to as PANoptosis—remain poorly understood. In this study, we reveal that infection with *Rickettsia heilongjiangensis* suppresses RIPK1 kinase-dependent apoptosis and necroptosis in human microvascular endothelial cells (HMEC-1). However, mitochondria-dependent apoptosis during the late stages of infection is essential for bacterial replication. Interestingly, inhibition of caspase-8 does not sensitize *Rickettsia*-infected host cells to necroptosis. Transcriptomic analysis further reveals that *Rickettsia* infection upregulates the host tumor necrosis factor (TNF) and NF-κB signaling pathways, which subsequently suppress RIPK1 kinase activity and contribute to the inhibition of host cell death. These findings provide new insights into the molecular mechanisms by which *Rickettsia* evades host defenses.

## INTRODUCTION

*Rickettsia* is a Gram-negative, obligate intracellular bacteria transmitted by hematophagous arthropods. Members of the genus *Rickettsia* were divided into four groups based on their phylogenetic characteristics: the ancestral group (AG), the typhus group (TG), the transitional group (TRG), and the spotted fever group (SFG) (1). One of the SFG members, *Rickettsia heilongjiangensis*, is the causative agent of Far-eastern spotted fever, one of the most severe tick-borne rickettsioses and the most commonly reported tick-borne infection that results in death in China (2–5). *Rickettsia* infections primarily cause pathology in the vasculature system, likely due to their strong affinity for vascular endothelial cells (ECs). Previous studies have shown that ECs are key immunoreactive cells involved in host defense and inflammation (6). Accordingly, rickettsial vasculitis, which is characterized by vascular inflammation, vascular permeability, and blood vessel damage, was caused by a series of rickettsial infections (7).

The human innate immune system recognizes invaded microbes and reacts rapidly by activating programmed cell death, including apoptosis, pyroptosis, and necroptosis (8). Pyroptosis and necroptosis are believed to be more inflammatory types of cell death that are triggered by the activation of the pore-forming proteins gasdermin D (GSDMD) and mixed lineage kinase domain-like protein (MLKL), respectively, in contrast to apoptosis, which is typically regarded as immunologically silent (9). Recent research showed that these cell death processes are highly interconnected by shared regulatory proteins and signaling pathways and are collectively termed PANoptosis (8). Upon stimulation by tumor necrosis factor (TNF), TRADD, RIPK1, and several ubiquitin ligases are recruited to the cytoplasmic death domain of the TNF receptor to initiate the formation of the signaling complex. Within the complex, rapid polyubiquitin on RIPK1 (receptor-interacting protein kinase 1) makes it a scaffold for recruiting a series of kinases, which subsequently activate the downstream NF-κB signaling pathways, leading to pro-survival gene activation. Dysregulation of this complex promotes the autophosphorylation and activation of RIPK1, which recruits and activates caspase-8, ultimately activating caspase-3 and leading to apoptosis, or cleaving GSDMD to induce pyroptosis. In instances where caspase-8 is inhibited, RIPK1 recruits RIPK3 to phosphorylate MLKL, thereby mediating necroptosis (10). As a result, RIPK1 and caspase-8 have been proposed as central regulators in the interplay of cell death pathways and the outcome of cell death development (11, 12).

Pathogens have evolved sophisticated mechanisms to modulate host cell death and facilitate survival, multiplication, and transmission. The interplay with host cell death progression would be more critical in determining the outcome of infection for the intracellular pathogen *Rickettsia*, which depends on its host to survive and propagate. Since a host cell that dies rapidly upon infection does not facilitate the growth of the intracellular pathogen, it is believed that the prevention of apoptosis may benefit from bacterial infection and reproduction in cases involving obligate intracellular bacteria (13–15). *R. rickettsi* was reported to protect infected vascular endothelial cells from apoptosis through the activation of the NF-κB signaling pathway, thereby inhibiting the activation of apical and effector caspases or controlling the intracellular levels and localization of pro- as well as anti-apoptotic proteins (16–18). As previously stated, multiple regulated cell death pathways were highly interconnected. However, the mechanisms underlying the development of apoptosis, pyroptosis, and necroptosis in endothelial cells (ECs) infected with *Rickettsia* remain poorly understood. Additionally, the intricate interactions between *Rickettsia* and these host cell death pathways have yet to be fully elucidated. Further research is needed to uncover how *Rickettsia* manipulates these pathways to establish infection and evade host immune responses, as well as how the balance between these cell death modalities influences disease progression and outcomes.

This study focused on the development of cell death in human microvascular endothelial cells (HMEC-1) following infection with *Rickettsia heilongjiangensis* strain B8 (*Rh*-B8), as well as the temporal relationship between bacterial replication and host cell responses. We observed that caspase-8 was inhibited throughout the infection, and the necroptosis pathway was also suppressed due to the inhibition of phosphorylation and activation of RIPK1, RIPK3, and MLKL, mediated by the TNFα and NF-κB signaling pathways. These findings suggest that targeting caspase-8 may not significantly influence the outcome of host cell death during *Rickettsia* infection. Furthermore, we discovered that the apoptosis observed during the late stages of infection is mitochondrion-dependent and is crucial for *Rh*-B8 replication in human microvascular endothelial cells (HMEC-1). These results expand our understanding of how SFGR interacts with host programmed cell death pathways, highlighting the pathogen’s ability to manipulate these processes to facilitate its survival and proliferation.

## RESULTS

### *Rh*-B8 infection induces host cell apoptosis *in vivo* and *in vitro*

**Fig 1:**
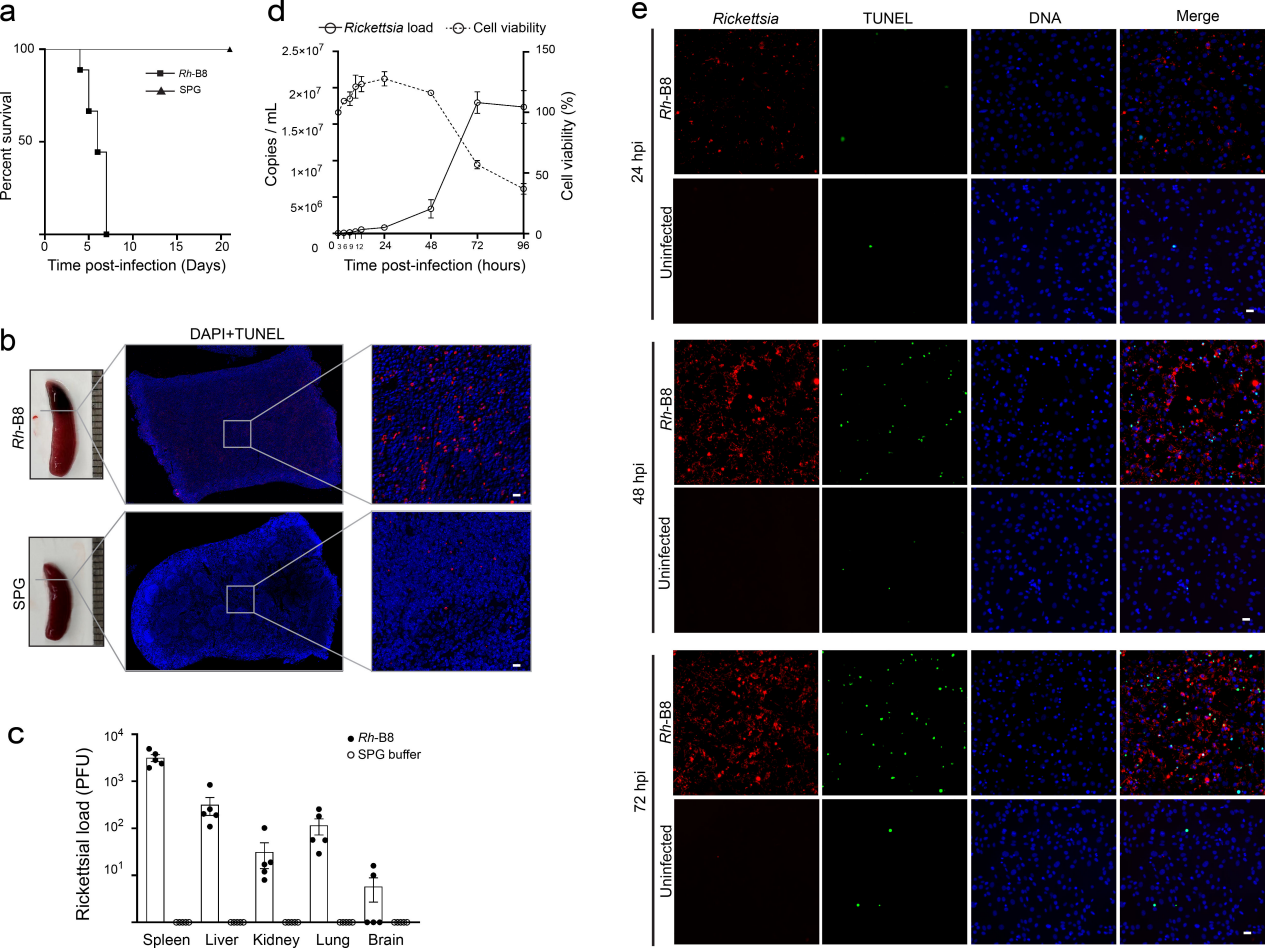
*Rh*-B8 infection-induced host cell apoptosis *in vivo* and *in vitro*. (a) Survival analysis of *Ifnar*^-/-^ mice infected with *Rh*-B8 (*n* = 9) compared to control mice injected with SPG buffer (*n* = 6). All mice died within one week following intravenous injection of *Rh*-B8. (b) Spleen tissue from *Rh*-B8-infected *Ifnar*^-/-^ mice exhibited enlargement and more severe pathological changes compared to uninfected mice. TUNEL staining on splenic paraffin sections revealed a multitude of apoptotic cells (red). Scale bar, 20 µm. Nuclei were indicated by blue DAPI staining. (c) Bacterial counts in tissues, including the spleen, liver, kidney, lung, and brain from five *Rh*-B8-infected *Ifnar*^-/-^ mice, were quantified using plaque assay. Means are shown with error bars representing ±SEM. (d) During infection at a multiplicity of infection (MOI) of 1.0, *Rh*-B8 exhibited slow propagation in the early stages of infection, followed by exponential growth starting at 48 hours post-infection (hpi). In parallel, the viability of HMEC-1 cells decreased significantly by 72 hpi. Data are expressed as mean ± standard deviation (SD), with each experiment conducted in biological triplicates, and results were averaged across all replicates. (e) Immunofluorescence microscopy images demonstrated an increase in the number of apoptotic HMEC-1 cells during *Rh*-B8 infection. Apoptotic cells were labeled using TUNEL staining (green), nuclei were visualized with DAPI (blue), and bacteria were detected using an anti-OmpB antibody (red). Scale bars, 20 µm.

To evaluate the pathogenicity of our laboratory-isolated *Rickettsia heilongjiangensis* strain B8 (*Rh*-B8) (4), we first assessed its virulence *in vivo* using type I interferon receptor-deficient mice (*Ifnar1-/-*) (19). All infected mice succumbed to *Rh*-B8 infection within one week of intravenous inoculation (Fig. 1a). Gross pathology showed extensive splenic necrosis in all succumbed mice (Fig. 1b). We next monitored bacterial loads across multiple organs in infected mice. Consistent with previous findings, splenic tissue showed the highest rickettsial burdens following intravenous *Rh*-B8 inoculation (Fig. 1c). To investigate the mechanism of tissue damage, terminal deoxynucleotidyltransferase-mediated dUTP-biotin nick end labeling (TUNEL) staining was performed on spleen sections from mice that died from infection. The results revealed a substantial number of apoptotic cells, indicating that apoptosis has a significant role in the observed pathology (Fig. 1b).

Programmed cell death, including apoptosis, pyroptosis, and necroptosis, can have dual roles in host–pathogen interactions. On one hand, these processes serve as a protective immune response to eliminate infected cells; on the other hand, excessive cell death can disrupt host homeostasis and exacerbate disease. To further elucidate the mechanisms of host cell death during rickettsial infection, we investigated the temporal progression of cellular damage induced by *Rh*-B8 infection in human microvascular endothelial cells (HMEC-1), which are known to be primary target cells of SFGR (20). Bacterial growth and host viability assays demonstrated that *Rickettsia* multiplies rapidly without causing significant host cell death until 72 hours post-infection (hpi) (Fig. 1d). TUNEL staining revealed a marked increase in apoptotic cells starting at 48 hpi (Fig. 1e). These results collectively demonstrate that *Rickettsia* infection induces host cell apoptosis both *in vivo* and *in vitro*.

### Transcriptome study revealed the activation of the TNF-signaling pathway

**Fig 2:**
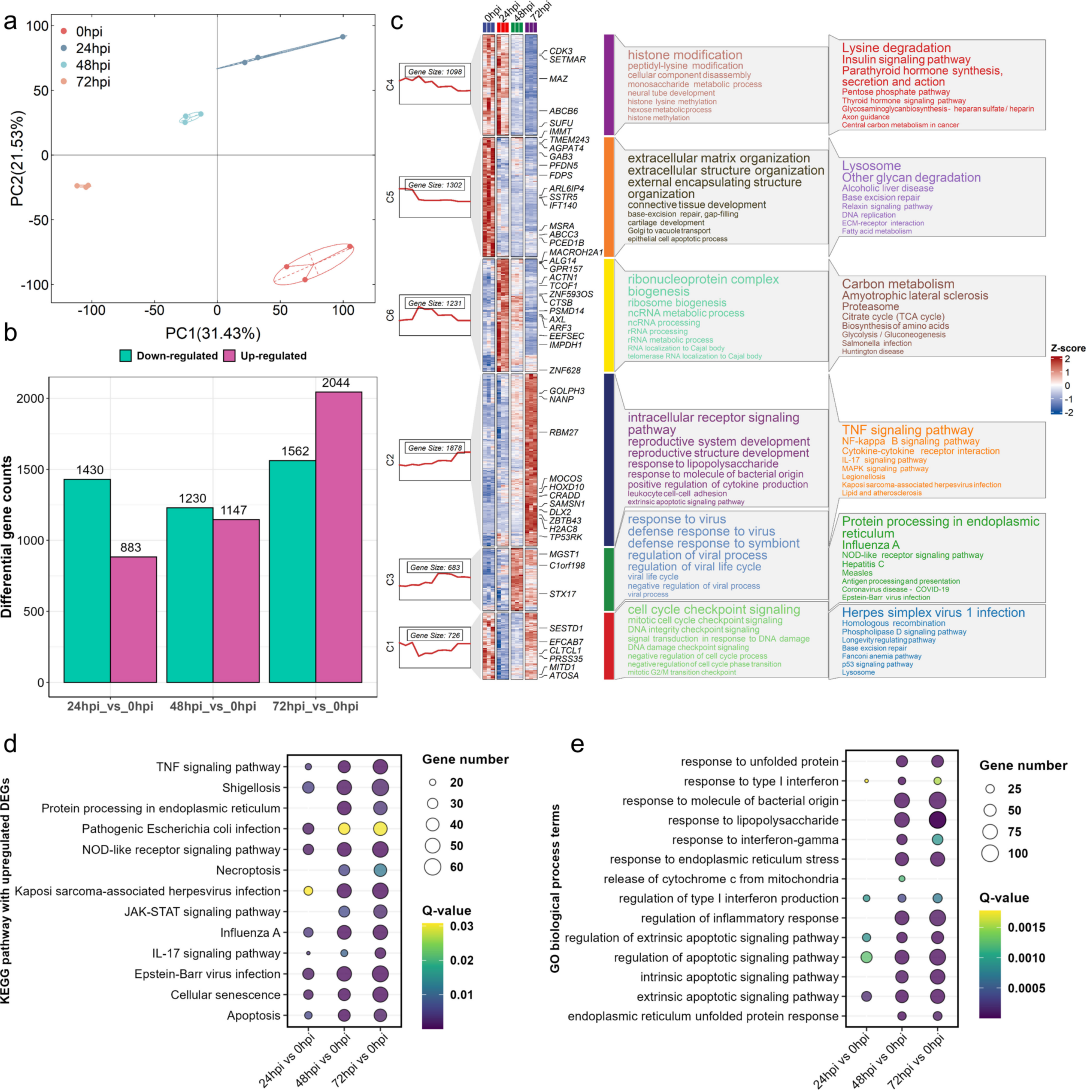
Transcriptomic analysis reveals genes and functional pathways disturbed by *Rh*-B8 infection. (a) The PCA score plot demonstrated a marked difference in the transcriptome profiles among cells infected with *Rh*-B8 at different time points compared to the control cells. (b) The histogram illustrates significant differences in the number of upregulated and downregulated genes between the various infection time groups and the control group. (c) An overall gene expression heatmap and temporal analysis were conducted. The figure is organized into nine columns, each representing distinct analytical layers. Column 1 (leftmost): a boxplot categorizing all differentially expressed genes (DEGs) into six distinct clusters, with temporal expression dynamics visualized across infection timepoints. Columns 2 to 5: heatmaps depicting gene-level expression patterns across experimental groups, with hierarchical clustering to emphasize shared regulatory trends. Column 6: focused visualization of genes of notable research interest, prioritized based on fold change and functional relevance. Column 7: color-coded bars representing functionally distinct gene sets. Column 8: Gene Ontology (GO) term enrichment analysis, highlighting biological processes significantly associated with DEG clusters. Column 9: Kyoto Encyclopedia of Genes and Genomes (KEGG) pathway enrichment analysis, identifying key signaling cascades perturbed during infection. (d) The bubble plot illustrates the KEGG enrichment analysis of upregulated genes at different infection time points in comparison to the control group. The size of the circles reflects the number of genes enriched in each pathway, whereas the color signifies the significance of the adjusted *q*-values. (e) Another bubble plot details the GO biological process terms enriched by upregulated genes at different infection time points compared to the control group. The size of the circles indicates the number of genes enriched in each pathway, while the color represents the significance of the adjusted *q*-values.

The dynamics of host gene expression determined by transcriptomics during the different stages of the *Rh*-B8 infection were investigated to assess the host cell damage mechanism. HMEC-1 cells were mock-infected or infected with the *Rh*-B8 strain at an MOI of 1.0 for 24, 48, and 72 hours, respectively. Principal component analysis (PCA) confirmed the reproducibility of each group and exhibited significant discrepancies between groups based on the transcriptomic gene profile (Fig. 2a). In total, compared to the mock-infected cell group, we found that the differentially expressed genes (DEGs) in cells at 24, 48, and 72 hpi were 2,313, 2,377, and 3,606, respectively. The numbers of up- and downregulated DEGs in 72 hpi were more than in 24 and 48 hpi, which may indicate that the interaction between *Rh-*B8 and host cells increased and reached the maximum at 72 hpi (Fig. 2b). To determine the dynamic response of HMEC-1 cells upon *Rh*-B8 infection, 5,672 differentially expressed genes among four groups were classified into six clusters using the K-means clustering method, considering their expression tendencies during the infection development (Fig. 2c). GO enrichment and KEGG pathway analyses were performed on these DEGs in each cluster. The results showed that genes grouped into clusters 4 and 5 were downregulated gradually with infection, while genes classified into cluster 2 and 3 were progressively upregulated. Furthermore, genes clustered together were more likely to be classified into the same functional gene set. For instance, DEGs in cluster 2 were significantly enriched in the “intracellular receptor signaling pathway” in biological process (BP), “TNF signaling pathway,” and “NF-κB signaling pathway” in KEGG pathways (Fig. 2c). To explore the patterns of host gene expression and functional pathways in response to *Rh*-B8 infection, we conducted a KEGG pathway enrichment analysis using differentially expressed genes (DEGs) from comparisons between various infection stages and mock infections. The KEGG analysis for each group showed that the upregulated DEGs were involved in pathways related to both apoptosis and necroptosis. (Fig. 2d). We further evaluated these DEGs for functional enrichment of GO term analyses along with the development of infection. The upregulated DEGs were mainly enriched to “response to unfolding protein,” “response to endoplasmic reticulum stress,” “intrinsic/extrinsic apoptotic signaling pathway,” and “endoplasmic reticulum unfolded protein response” (Fig. 2e).

### RIPK1-mediated apoptosis and necroptosis were suppressed during *Rh*-B8 infection

The TNF-mediated inflammatory signaling pathway is known to trigger RIPK1 kinase-dependent pyroptosis, apoptosis, and/or necroptosis (10). These interconnected cell death mechanisms, collectively termed PANoptosis, have been proposed to function as a robust host immune response against invasive pathogens (8). Transcriptome analysis revealed significant enrichment of the TNF-signaling pathway and genes associated with both necroptosis and apoptosis, suggesting their potential involvement during *Rickettsia* infection. While the TUNEL staining technique primarily detects apoptotic cells, the possibility of multiple cell death pathways being activated following rickettsial infection cannot be excluded. To investigate whether PANoptosis occurs in *Rickettsia*-infected cells, we conducted a series of blotting assays.

**Fig 3:**
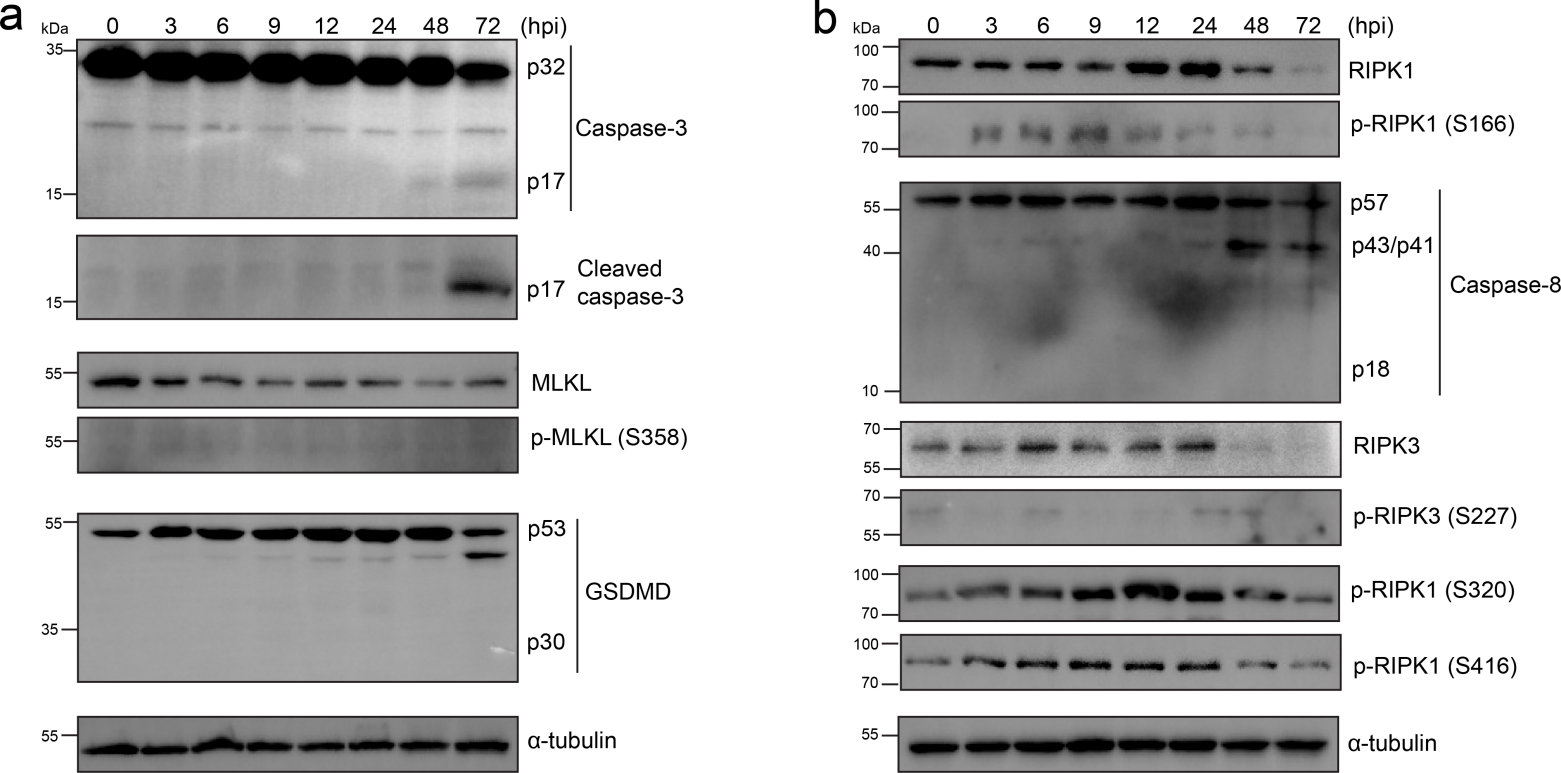
Inhibition of RIPK1-mediated apoptosis and necroptosis in HMEC-1. (a) Immunoblot assay shows increased levels of cleaved caspase-3 (P17) protein associated with apoptosis in *Rh*-B8-infected cells. HMEC-1 cells were infected with *Rh*-B8 and harvested at 3, 6, 9, 12, 24, 48, and 72 hpi. The levels of proteins associated with necroptosis and pyroptosis, including phosphorylated MLKL (S358) and cleaved GSDMD (P30), were not activated throughout infection. (b) Western blot assay indicates that auto-phosphorylated RIPK1 at Serine 166 was activated during early infection. Throughout the infection, RIPK1 did not activate cleaved caspase-8 (P18) or induce phosphorylation of RIPK3 (as indicated by phosphorylation at Serine 227). Instead, the upregulation of phosphorylation at Ser 320 and Ser 416 indicates the inhibition of RIPK1’s kinase activity. Representative results from three independent experiments are presented for the blotting assays described above.

The results showed that caspase-3 in HMEC-1 cells was processed into its active form only during the late stages of infection, and no phosphorylated MLKL (a marker of necroptosis) or activated GSDMD (a marker of pyroptosis) was detected (Fig. 3a). This suggests that apoptosis is likely the predominant form of cell death induced by *Rh-*B8 infection. Interestingly, auto-phosphorylation of RIPK1, a key initiator of RIPK1 signaling that promotes apoptosis and necroptosis (21), was observed as early as 3 hours post-infection (Fig. 3b). Caspase-8, which is recruited and activated by auto-phosphorylated RIPK1 to drive apoptosis, was only partially cleaved and not fully processed into its active form in *Rickettsia*-infected cells, even during the late stages of infection (Fig. 3b). Since caspase-8 inhibition can promote RIPK1-mediated necroptosis, we further examined the phosphorylation status of MLKL and RIPK3, which are recruited and phosphorylated by RIPK1 to execute necroptosis. However, no significant increase in their phosphorylation levels was detected during infection (Fig. 3a and b). Additionally, phosphorylation of RIPK1 at specific sites (Ser320 and Ser416) by kinases, such as IKKα/β, TAK1, and/or TBK1, has been shown to protect cells from RIPK1 kinase-dependent cell death (21). Our analysis revealed that phosphorylation at these sites increased immediately after infection (Fig. 3b), further supporting the inhibition of RIPK1-mediated cell death pathways. In summary, these findings indicate that RIPK1 kinase-mediated necroptosis and apoptosis are suppressed during *Rh-*B8 infection, despite partial activation of RIPK1.

### Inhibition of caspase-8 does not sensitize *Rh*-B8 infected HMEC-1 to necroptosis

**Fig 4:**
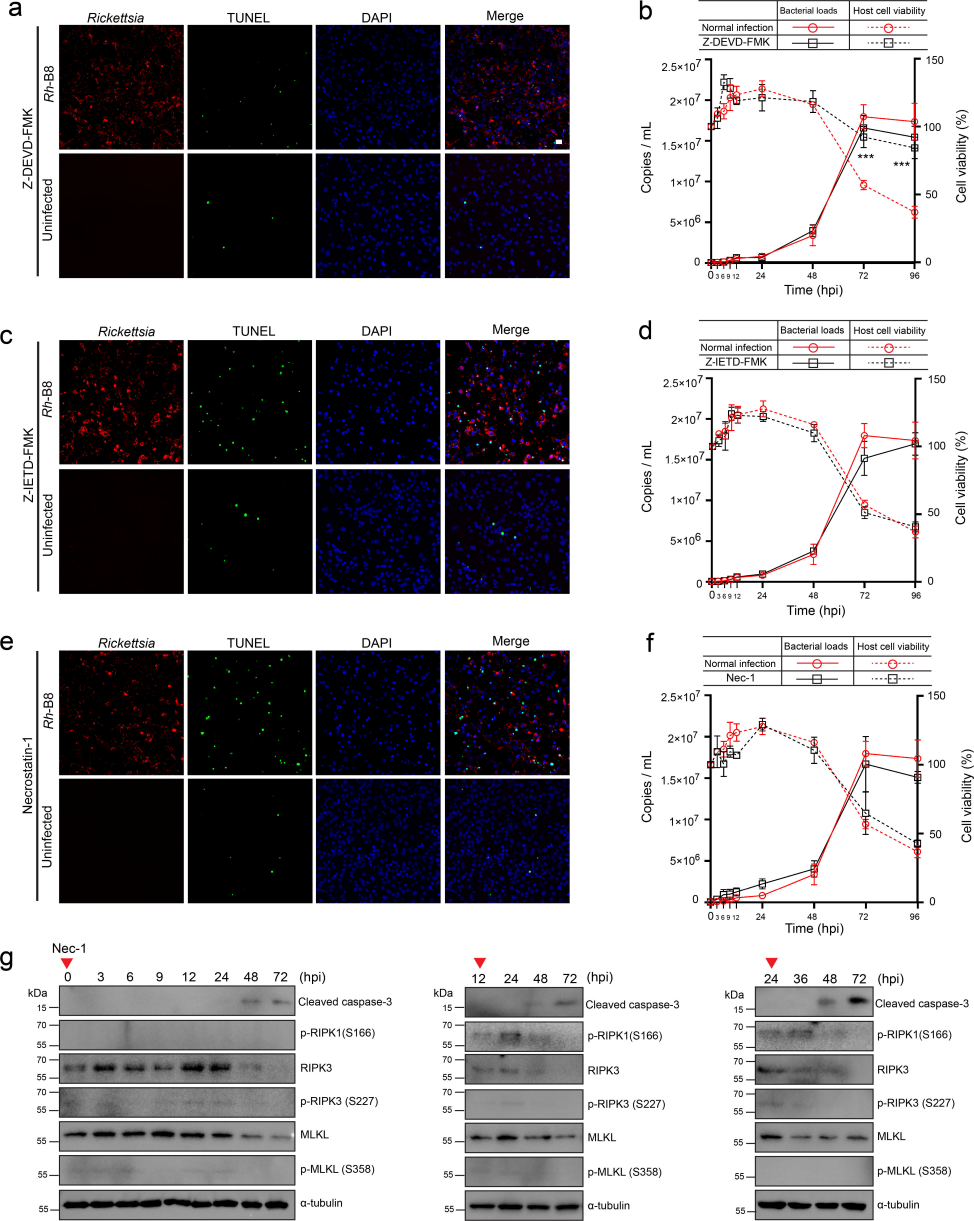
Inhibition of caspase-8 does not sensitize *Rh*-B8-infected HMEC-1 cells to necroptosis. (a and b) *Rh*-B8-infected cells treated with the caspase-3 inhibitor (Z-DEVD-FMK) showed a significant reduction in cell death during the late stages of infection. An immunofluorescence assay showcased images of TUNEL (green) and DAPI (blue) in HMEC-1 cells infected with *Rickettsia* (red) at 72 hpi. The growth curve illustrates the replication of *Rh*-B8 and cell viability of HMEC-1 treated or untreated with Z-DEVD-FMK. (c and d) *Rh*-B8-infected HMEC-1 cells treated with the caspase-8 inhibitor (Z-IETD-FMK) did not show protection against apoptosis at 72 hpi. The replication of *Rh*-B8 and cell viability of HMEC-1 between the Z-IETD-FMK-treated and untreated groups exhibited no significant difference. (e and f) The activity of RIPK1 kinase, blocked by necrostatin-1, also did not influence rickettsial growth or host cell viability. (g) The Western blot assay demonstrated that HMEC-1 cells infected with *Rh*-B8 and treated with the RIPK1 inhibitor necrostatin-1 (Nec-1) at various stages continued to undergo apoptosis. Representative results from three independent experiments are presented for the blotting assays. Uninfected cells treated with each inhibitor for 72 hours were included as controls in panels (a), (c), and (e). Data in (b), (d), and (f) were shown as mean ± SD (each time point has three biological replicates). ***P < 0.001 relative to normal infection, *P*-values were calculated using an unpaired *t*-test (two-tailed).

To test the hypothesis that *Rh-*B8 infection induces RIPK1-independent cell death, we evaluated the effects of inhibitors targeting key components of the RIPK1 signaling pathway. Inhibition of caspase-3 significantly reduced host cell death during the late stages of infection (Fig. 4a and b), consistent with the observation of apoptotic cells and the detection of fully processed caspase-3 after 48 hours post-infection (hpi). Caspase-8 is known to play a pivotal role in regulating host cell death following pathogen infection. When RIPK1 signaling is activated, caspase-8 typically initiates apoptosis; however, inhibition of caspase-8 allows RIPK1 to bind and phosphorylate RIPK3, leading to MLKL phosphorylation and subsequent necroptosis (11). In our study, caspase-8 was not fully activated at any point during *Rh-*B8 infection (Fig. 3b). Furthermore, inhibition of caspase-8 did not promote host cells to necroptosis, and apoptosis proceeded similarly to that observed in untreated infected cells (Fig. 4c and d). These findings suggest that caspase-8 plays a limited role in modulating host apoptosis and necroptosis during *Rh-*B8 infection and that alternative pathways may be driving the observed apoptotic cell death.

To further investigate the role of RIPK1, we used necrostatin-1 to inhibit RIPK1 kinase activity and assessed its impact on *Rh*-B8 infection. Inhibition of RIPK1 kinase activity did not alter bacterial growth or host cell survival (Fig. 4e and f). Additionally, blotting assays confirmed that RIPK1 inhibition at various stages of *Rh*-B8 infection did not prevent the eventual apoptosis of host cells (Fig. 4g). These results indicate that, although the TNF-signaling pathway and RIPK1 kinase-mediated cell death may be partially activated during *Rickettsia* infection, their progression to full execution of apoptosis or necroptosis is effectively blocked. This suggests that *Rickettsia* employs mechanisms to suppress RIPK1-dependent cell death pathways, ensuring host cell survival during the early and middle stages of infection while ultimately relying on RIPK1-independent apoptosis for host cell death in the late stages.

### RIPK1-mediated cell death was suppressed by NF-κB signaling activation

**Fig 5:**
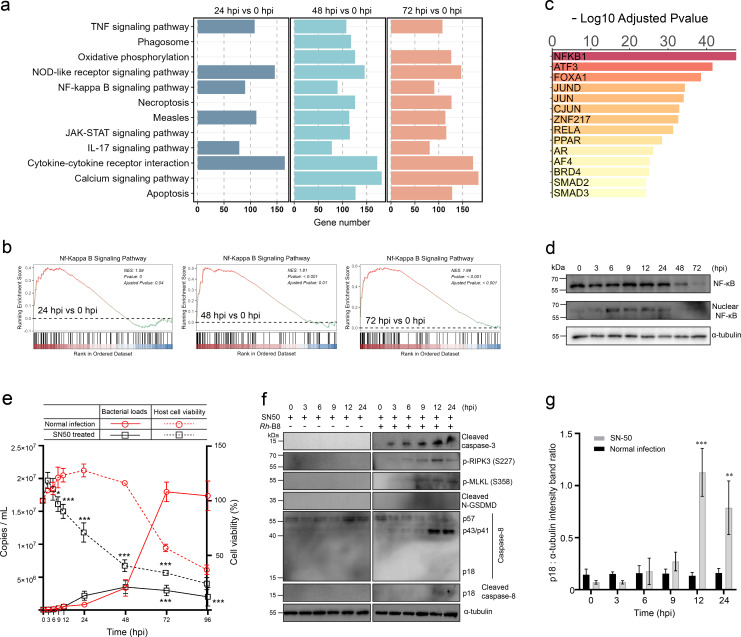
Nuclear factor κB protects against RIPK1-mediated host cell apoptosis and necroptosis during *Rh*-B8 infection. (a) The GSEA enrichment analysis, conducted based on the significantly upregulated genes between the infection group and the control group, indicates that the length of the bars represents the number of genes enriched in each pathway. (b) GSEA analysis indicates the NF-κB pathway was significantly associated with Rh-B8 infection at 24 hpi, 48 hpi, and 72 hpi. (c) The bar plot presents the top 14 significantly enriched transcription factors, with NFKB1 ranked as the primary contributor. The horizontal axis shows the −log_10_ adjusted *P*-value scale. (d) Immunoblot assay showcases the levels of total NF-κB in cell lysates and the amount that translocated into the nucleus. Representative results from three independent experiments are presented for the blotting assays. (e) Growth curves generated using quantitative RT-PCR at the indicated time points indicate that SN50 treatment, an inhibitor that restrains NF-κB translocation, significantly reduces cell viability and restricts *Rh*-B8 replication. *P < 0.1, ***P < 0.001 relative to normal infection, *P*-values were calculated using an unpaired *t*-test (two-tailed). (f) Western blot analysis was performed to evaluate the expression levels of key proteins, including cleaved caspase-3, phosphorylated RIPK3 (p-RIPK3, S227), phosphorylated MLKL (p-MLKL, S358), cleaved N-terminal GSDMD (N-GSDMD), caspase-8, and its cleaved isoforms, in HMEC-1 cells treated with SN50 upon infection by *Rh*-B8. Representative results from three independent experiments are presented for the blotting assays. (g) Densitometric analysis of cleaved caspase-8 (P18) during *Rh*-B8 infection, both with and without SN50 supplementation, was conducted using ImageJ. Statistical significance was assessed using Bonferroni’s multiple comparisons test. The data presented are representative of three biological replicates from three independent experiments. **P < 0.01, ***P < 0.001.

The stimulation of TNF signaling has been shown to promote the NF-κB pathway, which subsequently inhibits TNF-induced apoptosis (22). In this study, gene set enrichment analysis (GSEA) revealed that genes involved in NF-κB signaling were significantly upregulated immediately following *Rh*-B8 infection (Fig. 5a through c). To further investigate whether the NF-κB signaling pathway is upregulated, we performed a transcription factor prediction analysis. The results demonstrated significant enrichment of NF-κB-related genes, including NFKB1 (p50) and RelA (p65), suggesting that NF-κB is indeed likely activated (Fig. 5b). Consistent with this, blotting assays confirmed the accumulation of NF-κB in the host cell nucleus starting at 6 hours post-infection (hpi) (Fig. 5d).

To explore the functional role of NF-κB signaling in host cell survival during infection, an inhibitor of NF-κB translocation was introduced during infection. In the presence of the inhibitor, host cell viability declined rapidly, and rickettsial replication was significantly restricted, likely due to host cell exhaustion (Fig. 5e). These findings indicate that upregulation of NF-κB signaling is essential for maintaining host cell viability during *Rickettsia* infection. Next, we examined the progression of cell death following *Rickettsia* infection in the presence of the NF-κB inhibitor. The results showed that caspase-3 was processed into its active form immediately after infection, and phosphorylation of RIPK3 and MLKL could be detected during the early infection. However, no indicators of active GSDMD were observed (Fig. 5f). Additionally, fully activated caspase-8 could be detected when NF-κB translocation was inhibited, although it appeared later than cleaved caspase-3, suggesting that additional mechanisms may contribute to the execution of host cell apoptosis (Fig. 5f and g). Collectively, these results demonstrate that NF-κB signaling activation following *Rh*-B8 infection suppresses the progression of host cell apoptosis and necroptosis mediated by RIPK1.

### Mitochondrial apoptotic signaling contributes to the final host cell apoptosis

**Fig 6:**
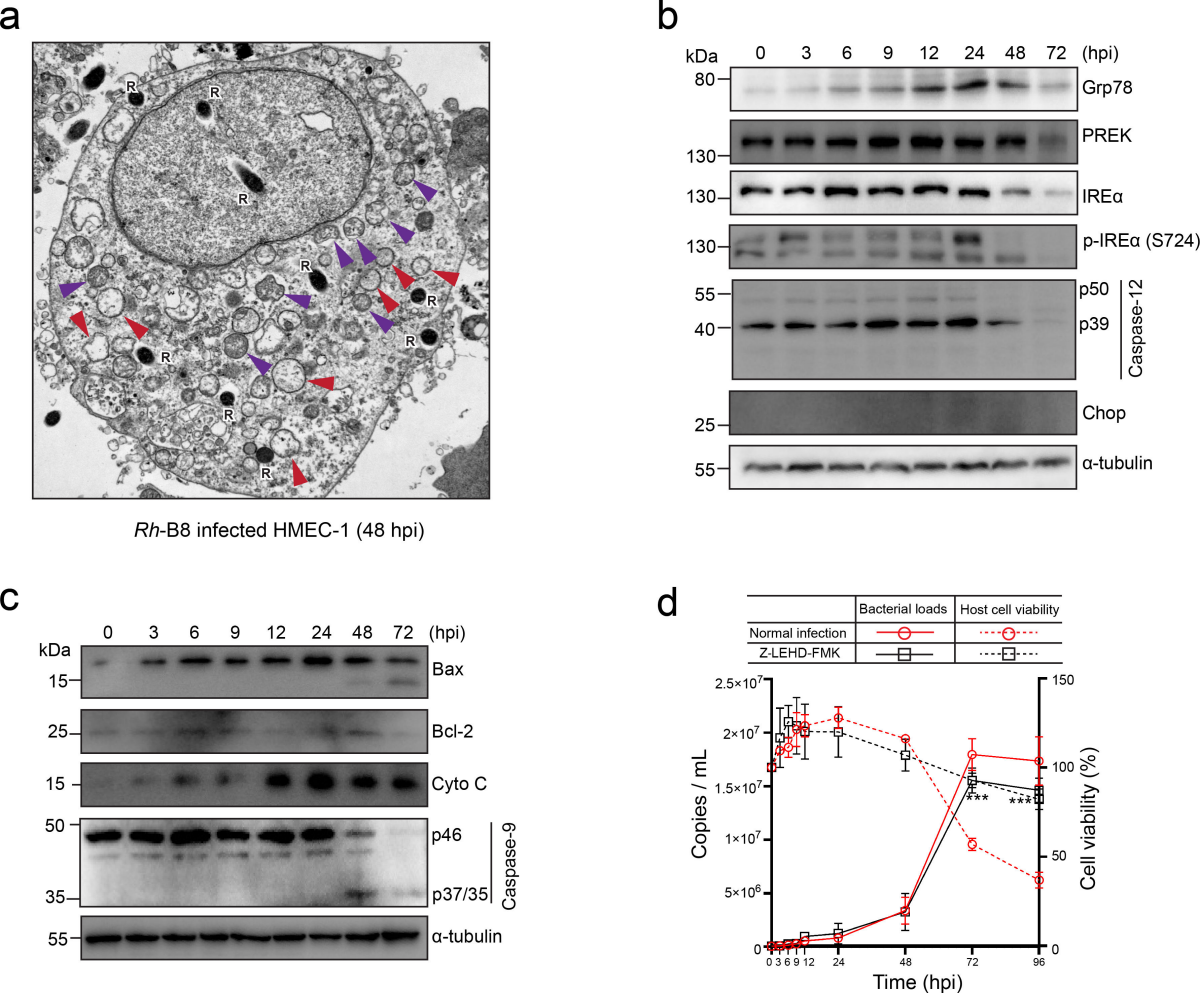
Mitochondrial apoptotic signaling plays a crucial role in the induction of host cell apoptosis. (a) Transmission electron microscopy images of HMEC-1 cells show perturbed morphology of cytoplasmic organelles infected with *Rh*-B8 at 48 hpi, including abnormal mitochondria characterized by swollen matrices and collapsed cristae (indicated with a purple triangle), as well as a disorganized endoplasmic reticulum exhibiting swollen morphology (indicated with a red triangle). The capitalized “R” represented *Rh*-B8 within the cell. (b) Immunoblot analysis shows bands of ER-stress-related proteins, including Grp78, PERK, IREα, p-IREα (S724), caspase-12, and CHOP in HMEC-1 cells infected with *Rh*-B8 at various time points. (c) Western blot analysis of proteins involved in mitochondrial pathway-mediated apoptosis, such as Bax, Bcl-2, Cyto C, pro (P46), and cleaved (p37 or p35) caspase-9, was conducted in HMEC-1 cells subjected to *Rh*-B8 infection at different stages. Representative results from three independent experiments are presented for the blotting assays. (d) Growth curves indicate that host cells treated with Z-LEHD-FMK (an inhibitor of caspase-9) exhibited significantly improved survival compared to control cells until 96 hpi, which corresponded with a decreased reproduction rate of *Rh*-B8. ***P < 0.001 relative to normal infection, *P*-values were calculated using an unpaired *t*-test (two-tailed).

Our transcriptome analysis revealed significant host endoplasmic reticulum (ER) and mitochondrial stress during *Rh*-B8 infection (Fig. 2e). Transmission electron microscopy (TEM) of *Rh*-B8-infected HMEC-1 cells further corroborated these findings, showing distinct intracellular morphological alterations, including dilatation of the rough-surfaced endoplasmic reticulum (RER) and the fragmentation and disappearance of mitochondrial cristae at 48 hours post-infection (Fig. 6a).

To explore the role of the intrinsic apoptotic signaling pathway in *Rh*-B8 infected HMEC-1 cells, we examined key molecular markers. We observed upregulation of Grp78 (Bip), IRE1α (inositol-requiring enzyme-1), and auto-phosphorylated IRE1α, all of which are involved in proper protein folding and the degradation of misfolded proteins (Fig. 6b). This upregulation confirmed the induction of ER stress and the activation of the unfolded protein response (UPR) in infected cells. However, during the late stages of infection, these proteins were degraded (Fig. 6b), suggesting a potential collapse of the UPR machinery. Notably, the active form of caspase-12, a mediator of ER stress-induced apoptosis, was absent during late infection and did not increase following infection. Additionally, no activation of C/EBP homologous protein (CHOP) was detected throughout the infection. These findings indicate that while ER stress and UPR were initiated, they may not have progressed sufficiently to trigger host cell apoptosis.

We then investigated mitochondrial stress and its contribution to apoptosis. A marked upregulation of Bax, a pro-apoptotic protein critical for mitochondrial-driven apoptosis, was observed (Fig. 6c). Furthermore, *Rh*-B8 infection led to a significant accumulation of cytochrome C released from mitochondria, indicative of increased mitochondrial permeability (Fig. 6c). Consistent with these observations, caspase-9 was fully activated during the late stages of infection. To assess the functional role of caspase-9 in *Rh*-B8-induced apoptosis, we employed a selective and irreversible caspase-9 inhibitor (Z-LEHD-FMK) during infection (Fig. 6d). Inhibition of caspase-9 activity significantly enhanced host cell survival up to 96 hours post-infection (hpi), supporting the hypothesis that mitochondrial stress-induced caspase-9 activation is a key driver of late-stage apoptosis during *Rh*-B8 infection. However, the inhibition of host cell apoptosis suppresses the growth of *Rh*-B8 during the late stages of infection. This suggests that host cell disruption in the later phase of infection might benefit the propagation and dissemination of *Rickettsia*.

## DISCUSSION

Programmed cell death is a fundamental mechanism employed by the human innate immune system to combat infections caused by intracellular pathogens. In turn, pathogens have evolved sophisticated strategies to delay host cell death and prolong cellular survival. Apoptosis, pyroptosis, and necroptosis are interconnected cell death pathways centrally regulated by caspase-8, all of which can be initiated through RIPK1 kinase signaling (12, 21). In this study, we demonstrate that during *Rh*-B8 infection, RIPK1 kinase-mediated apoptosis and necroptosis are suppressed. Moreover, we show that inhibiting RIPK1 or caspase-8 does not restrict *Rh*-B8 infection. Instead, the mitochondrial pathway drives the final stages of apoptosis in infected HMEC-1 cells, a process that may facilitate rickettsial dissemination between cells and enable the pathogen to evade host immune defenses.

Host cell death can be a critical determinant of disease outcomes during microbial infections. In our study, we found that mice lacking type I interferon (IFN-I) receptors succumbed to *Rh*-B8 infection via the vein injection. Additionally, the mice that died from *Rh*-B8 infection exhibited significant apoptosis in spleen tissues, which had the highest bacterial burden. Previous research has identified that type I interferon plays a crucial role in restricting the infection of *R. parkeri* (23). Moreover, our transcriptome analysis demonstrated significant enrichment of genes associated with the “interferon alpha response.” Therefore, during *Rh*-B8 infection, hosts lacking IFN-I receptors—which are essential for creating an inhospitable environment for obligate intracellular pathogens—are likely more susceptible to infection and rapid apoptosis.

Our transcriptome analysis revealed a significant upregulation of genes associated with the TNF signaling pathway. Consistent with this finding, the enrichment of downstream MAPK and NF-κB signaling pathways further corroborates the activation of TNF signaling upon *Rh*-B8 infection. Unlike previous studies that utilized TNF stimulation to investigate RIPK1-mediated cell death, our research focuses on elucidating the host’s intrinsic TNF signaling response during rickettsial infection.

The serine/threonine kinase RIPK1, recruited by TNFR1 upon TNF sensing, has emerged as a pivotal regulator of inflammatory responses, modulating various downstream immune receptors. Intriguingly, RIPK1 exhibits dual functionality: it can act as a scaffold protein to prevent cell death or as an active kinase to promote cell death. As a scaffold molecule, RIPK1 facilitates gene transcription by promoting the activation of NF-κB and MAPK signaling pathways, which collectively enhance the expression of pro-survival and inflammatory genes, thereby protecting cells from apoptosis and necroptosis. Conversely, as an active kinase, RIPK1 can paradoxically induce apoptosis, necroptosis, or pyroptosis (10). The activation and functional switch of RIPK1 is finely regulated by a series of phosphorylation events. Specifically, autophosphorylation of RIPK1 at Ser14/15, Ser20, Ser161, and Ser166 is indicative of its kinase activity, which can subsequently lead to necroptosis or apoptosis (24). Moreover, the kinases IKKα/β and TBK1/IKKε, recruited by the TNFR complex, phosphorylate RIPK1 at distinct sites, thereby inhibiting RIPK1-dependent cell death mediated by its kinase activity (25). During *Rh*-B8 infection, we observed autophosphorylation of RIPK1 at Ser166, alongside phosphorylation at Ser320 and Ser416, sites known to be targeted by IKKα/β and/or TBK1/IKKε to suppress RIPK1 kinase activity. This suggests that while RIPK1 kinase activity may be partially activated in the early stages of infection, the recruitment of inhibitory kinases ultimately prevents RIPK1-mediated host cell death.

A recent study on *Coxiella burnetii*-infected bone marrow-derived macrophages (BMDMs) identified caspase-8 as a critical regulator of bacterial replication by modulating host TNFα production and suppressing apoptosis (26). However, in *Rh*-B8 infection, caspase-8 remained inactive throughout infection, with no evidence of GSDMD cleavage, a process associated with pyroptotic cell death. Consistent with these observations, inhibition of either RIPK1 or caspase-8 did not alter the outcomes of cell death or bacterial growth. In contrast, NF-κB inhibition triggered rapid cell death, which was characterized by a combination of necroptosis and apoptosis.

Previous research on extracellular pathogens, such as *Yersinia pseudotuberculosis*, enteropathogenic *Escherichia coli* (EPEC), and enterohemorrhagic *Escherichia coli* (EHEC), demonstrated that bacterial effectors like YopJ and NleE acetylate and antagonize TAK1, IKKα/β, and MAPK kinases (MAPKKs), thereby limiting NF-κB and MAPK-driven pro-inflammatory cytokine expression and promoting RIPK1-dependent caspase-8 activation, which is crucial for controlling bacterial infection and dissemination *in vivo* (27, 28). Similarly, intracellular enteric pathogens, such as *Shigella flexneri* and *Salmonella typhimurium*, secrete effectors like OspZ and AvrA to inactivate NF-κB signaling (29). However, our findings in *Rh*-B8 infection contrast with these observations, highlighting the need for further investigation into how *Rh*-B8 and its effectors recruit kinases to repress RIPK1 kinase activity and restrict caspase-8 activation.

The release of cytochrome c and the processing of caspase-9 during the late stages of *Rh*-B8 infection indicate that the intrinsic apoptotic pathway is responsible for the eventual cell death. This mitochondrion-dependent apoptosis has also been documented in human cells infected with *R. rickettsii* and tick cells infected with *R. parkeri* (18, 30). These findings suggest that mitochondria-dependent apoptosis may represent a conserved cellular response to rickettsial infections. Given that *Rickettsia* relies on the host for survival, it is likely that the pathogen maximizes the utilization of host resources, including mitochondrial energy, until the host is depleted. This aligns with our observations, where significant apoptosis was only detected during the late stages of infection. The inhibition of caspase-3 and caspase-9 significantly enhanced host cell viability during late infection, coinciding with the termination of *Rh*-B8 replication (Fig. 4b and 6d). We hypothesize that while early-stage apoptosis suppression promotes bacterial survival and replication, delayed induction of regulated cell death may facilitate bacterial egress from infected host cells, thereby enabling systemic dissemination to adjacent cells or new hosts. Paradoxically, sustained host cell viability may limit bacterial transmission efficiency, a phenomenon consistent with observations in *Salmonella* and *Shigella* infections (31, 32). Elevated cellular viability may further potentiate host defense mechanisms, including autophagy induction (33) and metabolic competition for essential nutrients (e.g., iron, glucose), alongside the generation of antimicrobial metabolites such as reactive oxygen species (34). These multifactorial pressures collectively establish a restrictive microenvironment that suppresses *Rh*-B8 proliferation during late infection. As an obligate intracellular pathogen, *Rickettsia* employs a biphasic survival strategy: transient inhibition of apoptosis during initial infection transitions to regulated cell lysis in later phases. This temporally coordinated mechanism likely reflects an evolutionarily refined adaptive strategy to balance intracellular persistence and intercellular propagation, ensuring optimal bacterial fitness across distinct stages of the host–pathogen interaction.

Since the increase of cytochrome c release is observed to occur early in the infection process, while the manifestation of apoptosis is late, further investigation is needed to determine whether *Rickettsia* can modulate mitochondrial permeability to prevent premature cell death. The early release of cytochrome c suggests that mitochondrial integrity is compromised during the initial stages of infection. However, the delayed onset of apoptosis implies that *Rickettsia* may employ mechanisms to temporarily counteract or regulate this process, ensuring host cell survival during the critical early phase of bacterial replication. Understanding whether and how *Rickettsia* influences mitochondrial permeability could provide valuable insights into its strategies for evading host cell death and optimizing its intracellular lifecycle. Transmission electron microscopy (TEM) revealed notable mitochondrial damage and the presence of phagosomes, raising the question of whether *Rh*-B8 infection induces mitophagy to eliminate damaged mitochondria and thereby delay mitochondria-dependent apoptosis. Additionally, *Rh*-B8-infected host cells exhibited swollen and rounded endoplasmic reticulum (ER) structures, suggesting the induction of ER stress. However, this stress did not progress to full-blown apoptosis. The effector protein RARP-2, secreted by *R. rickettsii* via its type IV secretion system, has been proposed to interact with and alter the structure of the host cell’s ER (35). Nevertheless, the precise mechanisms by which rickettsial effectors interact with and disrupt ER-dependent apoptotic signaling pathways remain to be elucidated. Further studies are essential to clarify how *Rickettsia* manipulates host cell processes to delay apoptosis and ensure its survival.

## MATERIALS AND METHODS

### *Rickettsia heilongjiangensis* B8 strain, purification and infection

The *R. heilongjiangensis* B8 strain was isolated and preserved by our laboratory (4). Bacteria were propagated in Vero E6 cells and purified from cytoplasmic lysates as previously described (4). Briefly, cells infected for seven days were scraped and transferred into a 50 mL centrifuge tube. The suspension was centrifuged at 12,000 × *g* for 20 minutes, and the supernatant was discarded. The pellet was resuspended in 10 mL of phosphate-buffered saline (PBS) and mixed with an equal volume of 0.3 mm glass beads. The mixture was vortexed for 30 seconds, followed by incubation on ice for 30 seconds. This process was repeated three times, with the supernatant carefully transferred to a new centrifuge tube after each cycle. An additional 10 mL of PBS was added, and the disruption process was repeated, with the supernatant collected again. This step was performed three times. The resulting suspension was centrifuged at 400 × *g* for 30 minutes, and the supernatant was transferred to a new tube and centrifuged at 12,000 × *g* for 30 minutes. The final pellet, containing the preliminarily purified bacteria, was resuspended in SPG solution (0.218 M sucrose, 3.76 mM potassium dihydrogen phosphate, 7.1 mM dipotassium hydrogen phosphate, 4.9 mM glutamine, and 10 mM magnesium chloride) and stored in aliquots at −80°C.

For the infection study, human microvascular endothelial cells (HMEC-1) were used to investigate the cellular responses and molecular mechanisms following *Rh*-B8 infection at different time points. HMEC-1 cells were cultured in endothelial cell medium (ECM, ScienCell, San Diego, USA) supplemented with 10% fetal bovine serum (FBS), 100 U/mL penicillin, and 100 µg/mL streptomycin at 37°C with 5% CO₂. Cells were suspended at a density of 1 × 10^7^ cells/mL in ECM containing 10% FBS and seeded into 10 cm plates (*n* = 3 per group). When the cells reached approximately 70–80% confluence, *Rh*-B8 was introduced at a multiplicity of infection (MOI) of 1.0 (36). To perform the transcriptome analysis, infected cells were harvested at 0, 24, 48, and 72 hours post-infection (hpi) by scraping, with each sample containing approximately 10^7^ cells. The 0 hpi samples served as mock-infected controls and were designated the control group. The remaining groups were labeled Model1, Model2, and Model3, corresponding to 24, 48, and 72 hpi, respectively.

### Transcriptome analysis

Total RNA was extracted from *Rh*-B8-infected cells or mock-infected cells using TRIzol Reagent according to the manufacturer’s instructions. To prepare the cDNA library, total RNA was treated with RNase-free DNase I. Then, mRNA was purified using magnetic oligo (dT) beads and evaluated using the Agilent 2100 bioanalyzer (Agilent Technologies, Santa Clara, CA, USA) for RNA integrity. mRNAs with RNA integrity numbers (RINs) > 8 were subjected to subsequent analysis. The purified mRNA was used to construct libraries using TruSeq PE Cluster Kit v3-cBot-HS (Illumina, San Diego, CA, USA) according to the manufacturer’s instructions. Then, these libraries were sequenced on an Illumina HiSeq platform (Illumina, USA) at BioNovoGene Tech. Co. (Suzhou, China). The raw data of RNA-seq have been deposited with links to BioProject with accession number PRJNA1221310 in the NCBI BioProject database (https://www.ncbi.nlm.nih.gov/bioproject/). Quantification of gene expression levels was estimated by the fragments per kilobase of transcript per million fragments (FPKM) mapped method. Differential expression analysis of two samples was performed using the DESeq R package. The *P*-values were adjusted by FDR (false discovery rate). An FDR < 0.05 and | log2 FC (fold change) | ≥1 were set as the thresholds for significant differential expression. Gene Ontology (GO) enrichment and Kyoto Encyclopedia of Genes and Genomes (KEGG) pathway analyses of DEGs were conducted according to the protocols described by previous reports (37, 38).

### Mouse experiment

All mice used in this study were knockout strains lacking the genes encoding type I interferon receptors (*Ifnar*1^−/−^) on a C57BL/6J background (19). The mice were housed in BSL-3 laboratories at the State Key Laboratory of Pathogens and Biosecurity, Academy of Military Medical Sciences, in compliance with protocols approved by the Ethics Committee of Anhui Medical University (No. LLSC20200351) and in accordance with the Chinese National Guidelines for the Care of Laboratory Animals and Institutional Animal Care. To estimate live bacteria inoculated in the mouse study, the plaque assay was performed in Vero cells as previously described (39). For infection, each mouse was intravenously injected with 1 × 10⁵ PFU of bacteria. Briefly, the tail was sterilized with 75% ethanol, and 200 µL of bacterial suspension was administered using a 30.5-gauge needle into the lateral tail vein. To assess the susceptibility of *Ifnar1*^−/−^ mice to *Rh*-B8 infection, nine mice were intravenously inoculated and monitored for survival. All infected mice succumbed within one week post-infection. Immediately after death, tissue samples (spleen, lung, liver, and brain) were collected from *Rh*-B8-infected *Ifnar1*^−/−^ mice, photographed, and processed for histological analysis by paraffin embedding and sectioning (5 µm thickness). Apoptotic cells in tissue sections were detected using the TUNEL assay (Procell One-step TUNEL Apoptosis Assay Kit) according to the manufacturer’s protocol. Fluorescence imaging was performed at 40 × magnification using a THUNDER Imaging System (Leica). For bacterial burden quantification, five infected mice showing severe clinical signs (significant hypothermia and profound lethargy impairing normal mobility) by day 5 post-infection were humanely euthanized under isoflurane anesthesia. Organ samples were collected for subsequent plaque analysis as described previously (39). Briefly, each organ tissue was homogenized in 1.0 mL of normal saline, and 10-fold serial dilutions were plated on Vero cell monolayers. Plaques were counted by visual inspection between 10 and 14 days post-infection.

### Bacterial growth analysis

The number of *Rh*-B8 in infected HMEC-1 cells was estimated by quantitative real-time PCR. Mock-infected or *Rh*-B8-infected HMEC-1 cells were collected at indicated times, and the genomic DNA was extracted following the Genomic DNA Extraction Kit (TaKaRa, 9765), according to the manufacturer’s protocols. The integrity of the genomic DNA was examined and then analyzed by quantitative real-time PCR targeting the *Rickettsia*-specific outer membrane protein B (ompB) gene, using primers ompB-F (forward-CATCAGTATAGAAAGGTTTTGCCCATA) and ompB-R (reverse- ATCTGAAGCGGGAGCAATACC). Quantitative real-time PCR was performed using the Premix Ex Taq (TaKaRa, RR390) on a quantitative real-time PCR detection device (Roche, LightCycler 96). The relative abundance of *Rickettsia* was calculated by the 2^-△△Ct^ method.

### Cell viability assay

Cell activity was analyzed by Cell Counting Kit-8 (GOONIE, 100-106) according to the manufacturer’s protocols. HMEC-1 cells were seeded into 96-well microplates (Corning, 3599). After *Rh*-B8 infection and inhibitor treatment, 10 µL of CCK-8 reagent was added to each well and then incubated for 1.5 hours at 37 ℃. The absorbance was analyzed at 450 nm using an absorbance microplate reader (MOLECULAR DEVICES, CMAX PLUS). The activity of HMEC-1 cells was expressed by the values of absorbance.

### Caspase and kinase inhibition studies

HMEC-1 cells were plated 24 hours prior to infection. The next day, diluted inhibitors were added to an ECM culture medium containing 2% FBS, followed by the addition of bacteria for infection. The six-well plates were then placed in a 34°C incubator with 5% CO_2_. Cells were collected at the indicated times and then used for the quantification of cell viability and bacterial growth. The following inhibitors were used: Z-DEVD-FMK, a caspase-3 inhibitor, was purchased from MedChemExpress (HY12466) and used at a working concentration of 50 µM. Z-IETD-FMK, a caspase-8 inhibitor (MedChemExpress, HY-101297), was used with a working concentration of 30 µM. Necrostain-1, a RIPK1 inhibitor (MedChemExpress HY-15760), was used at 25 µM. SN50, an NF-κB translocation inhibitor (MedChemExpress HY-P0151), was used at a working concentration of 25 µM. Lastly, Z-LEHD-FMK, a caspase-9 inhibitor (MedChemExpress HY-P1010), was used at a concentration of 30 µM.

### Western blot

After *Rh*-B8 infection, the supernatants were removed at the indicated time points. HMEC-1 cells were washed twice with 1 × PBS and then lysed with 2 × lysis buffer (Tris-HCl, Glycerol, SDS, Bromophenol blue, B-mercaptoethanol). The protein samples from HMEC-1 cells were boiled at 100℃ for 10 minutes. Proteins were separated by SDS-PAGE using different concentrations of polyacrylamide gels. After performing SDS-PAGE, the separated proteins were transferred onto polyvinylidene difluoride membranes (Millipore, ISEQ00010), and then, nonspecific binding was blocked by incubation with 5% skim milk in the TBST for 2 hours at room temperature (RT). Proteins on the membranes were detected with primary antibodies against anti-cleaved-caspase-3 (Cell Signaling, 9664, 1:1000), anti-caspase-3 (Cell Signaling, 14220, 1:1000), anti-caspase-8 (Cell Signaling, 9746, 1:1000), anti-caspase-9 (Cell Signaling, 9502, 1:1000), anti-caspase-12 (Proteintech, 55238-1-AP, 1:1000), anti-GSDMD (Cell Signaling, 69469, 1:1000), anti-cleaved N-terminal GSDMD (Abcam, ab215203, 1:1000), anti-phospho-MLKL S358 (Abcam, ab187091, 1:1000), anti-MLKL (Cell Signaling, 14993, 1:1000), anti-phospho-RIPK3 S227 (Cell Signaling, 93654, 1:1000), anti-RIPK3 (Cell Signaling, 10188, 1:1000), anti-phospho-RIPK1 S166 (Cell Signaling, 65746, 1:1000), anti-phospho-RIPK1 S320 (Cell Signaling, 39341, 1:1000), anti-phospho-RIPK1 S416 (Cell Signaling, 71420, 1:1000), anti-GRP78 (Abcam, ab108615, 1:5000), anti-PERK (Cell Signaling, 5683, 1:1000), anti-phospho-IREα S724 (Abcam, ab124945, 1:1000), anti-IREα (Cell Signaling, 3294, 1:1000), anti-CHOP (Cell Signaling, 2895, 1:1000), anti-Bax (Cell Signaling, 5023, 1:1000), anti-Bcl-2 (Cell Signaling, 15071, 1:1000), anti-cytochrome c (Cell Signaling, 11940, 1:1000), and anti-NF-κB (Proteintech, 66535-1-Ig, 1:2000). The membranes were then washed three times with TBST for 30 minutes and further incubated with the horse-radish peroxidase (HRP)-conjugated secondary antibodies for 1.5 hours at RT. The membranes were developed with chemiluminescence and imaged using a chemiluminescence imaging system (Clinx, ChemiScope 6000) to visualize the protein bands.

### Immunofluorescence assay

The day before infection, 15 mm cell coverslips were placed in a 12-well plate, and digested HMEC-1 cells were added. The plates were then incubated in a 37°C incubator with 5% CO_2_. The following day, cells were infected with *Rh*-B8 at an MOI of 1.0. At the indicated time point, the coverslips were collected and fixed at RT for 30 minutes using 4% paraformaldehyde (Beyotime, P0099). Next, the cells were treated with a permeabilization solution (Beyotime, P0095) for 10 minutes at RT. This was followed by a 15-minute blocking step using a blocking solution (Beyotime, P0260). For each coverslip, 100 µL of TdT Equilibration Buffer (Procell, P-CA-301) was added, and the samples were incubated at 37°C for 20 minutes. After removing the buffer, 50 µL of labeling solution (which included a 1:500 dilution of custom-made ompB antibody) was added. Subsequently, 50 µL of a 1:400 dilution of goat anti-rabbit antibody (Abcam, ab150080) was added. Finally, the cell side of the coverslip was inverted onto a microscope slide containing a DAPI anti-fade reagent (Beyotime, P0131). The samples were observed and photographed using a fluorescence microscope (Thunder), with apoptotic cells viewed at 488 nm, bacteria at 594 nm, and cell nuclei at 380 nm.

### Transmission electron microscopy

Cultured cells were fixed with 2.5% glutaraldehyde in PBS for 1 hour at room temperature. Subsequently, post-fixation was performed using 1% osmium tetroxide (Electron Microscopy Sciences, Hatfield, PA, USA) containing 1% potassium ferricyanide (Thermo Fisher). The samples underwent a series of alcohol dehydration steps with gradually increasing concentrations, followed by three treatments with epoxy resin at each step, each lasting 1 hour. After removal of the epoxy resin, resin-filled beam capsules were inverted over the cells and incubated overnight at 37°C, followed by an additional 48-hour incubation at 60°C. The cells were examined and photographed at 80 kV using a Jeol 1011 transmission electron microscope (Jeol, Peabody, MA, USA).

### Statistical analyses

All statistical analyses were conducted in the R platform (version 4.0) or with GraphPad Prism (version 9.5.0). For statistics in multiple groups, we utilized Kruskal–Wallis one-way ANOVA to evaluate the differences among the three groups. Only the remarkably different indices in the three groups were evaluated by further Mann–Whitney U test with Bonferroni correction as a post-hoc test between each of the two groups. Values of adjusted *P* value less than 0.1 were considered statistically significant. Error bars represent the mean ± SEM or mean ± SD as indicated. The Mfuzz package in R was used to conduct the cluster analysis. Spearman’s rank correlation coefficients were calculated and corrected for multiple testing using the Benjamini-Hochberg method. Principal component analysis (PCA) was performed by using the *dudi.pca* function in the R package ‘ade4’.
